# Building resilience against cholera: lessons from the implementation of integrated community strategy for cholera control in Zambia

**DOI:** 10.1136/bmjgh-2024-017055

**Published:** 2025-01-22

**Authors:** Moses Mwale, Peter Jay Chipimo, Precious Kalubula, Ladislas Hibusu, Stella Mumba Chomba Mulima, Kafusha Kapema, Kelvin Mwangilwa, Nyuma Mbewe, Fred Kapaya

**Affiliations:** 1 World Health Organization, Lusaka, Zambia; 2 Zambia National Public Health Institute, Lusaka, Zambia; 3 World Health Organization, Nairobi, Kenya

**Keywords:** Cholera, Health education and promotion, Hygiene, Public Health

## Abstract

Cholera has remained a persistent public health challenge in Zambia since the country’s first reported outbreak in 1977. The recent outbreak, which began in October 2023 and is ongoing as of June 2024, is the most severe in Zambia’s history and part of the larger 2022–2024 Southern Africa cholera outbreak, which has affected multiple countries in the region. This article describes the implementation of the integrated community strategy for cholera control (ICSCC) in three districts of the Copperbelt Province during this outbreak. The ICSCC is a comprehensive, community-centric public health approach that integrates surveillance, case management, water, sanitation and hygiene interventions, community engagement and infection prevention measures. The strategy’s implementation involved deploying multidisciplinary technical teams, training community-based volunteers and healthcare workers in the affected communities. This approach led to a rapid reduction in cholera cases and mortality, largely due to enhanced surveillance, community education sessions and improved sanitation practices. The ICSCC also improved stakeholder coordination and enabled rapid communication for early response to cholera hotspots. Key lessons learnt include the importance of robust coordination, early community involvement and context-specific adaptations. The strategy’s emphasis on data-driven decision-making and adaptation to local socio-cultural dynamics was crucial for its effectiveness. These findings underscore the potential of integrated community-based approaches in managing cholera outbreaks, enhancing public health preparedness and building long-term resilience. The ICSCC strategy offers a scalable model for regions facing similar public health challenges, providing valuable insights for policymakers and practitioners on the effectiveness of community involvement in managing public health crises.

**Summary box**

- Problem: The 2023–2024 cholera outbreak in Zambia was the largest in the nation’s history, with over 23 000 reported cases and 739 deaths, resulting in a case fatality rate of 3.2%.
- Intervention: The integrated community strategy for cholera control was implemented in the three most affected districts of the Copperbelt Province, focusing on surveillance, case management, water, sanitation and hygiene interventions, community engagement and infection prevention and control.
- Impact: The strategy led to a notable decrease in number of new cases and community deaths, improved prevention practices and strengthened local response mechanisms in Ndola, Kitwe and Chililabombwe.
- Lessons Learnt: Effective cholera management requires strong stakeholder coordination, early community involvement and adapting strategies to local risks. Integrated community approaches are promising for outbreak control and public health preparedness.

## Introduction

 Cholera, an acute diarrhoeal disease caused by the ingestion of *Vibrio cholerae*, remains a significant public health challenge worldwide. It is characterised by severe dehydration and can be fatal if untreated.^1^ Cholera outbreaks often occur in settings with inadequate access to clean water and sanitation, disproportionately affecting vulnerable populations such as children, pregnant women, the elderly and those living in poverty-stricken areas or unplanned settlements.^2 3^ The disease poses a major public health threat, leading to substantial morbidity, mortality and economic burden, particularly in developing countries.^4^,^6^

In Zambia, cholera has been a recurring public health crisis since its first recorded outbreak in 1977–1978. Notable outbreaks in 1991, 1992 and 1999 cumulatively accounted for over 38 000 cases and nearly 2300 deaths.^7^,^9^ The 2017–2018 outbreak marked a significant escalation, with an attack rate of 35.2 per 100 000 population, 5905 suspected cases and 114 deaths (case fatality rate (CFR): 1.9%) across seven provinces, with Lusaka Province contributing 92% of cases.^10 11^ This outbreak catalysed the development of Zambia’s Multisectoral Cholera Elimination/Control Plan, aiming to address systemic vulnerabilities contributing to recurrent cholera outbreaks.^11^

The most recent cholera outbreak in Zambia, spanning October 2023 to June 2024, emerged as the most severe and largest in the nation’s history, with 23 277 cases and 740 deaths (CFR: 3.2%) reported as of 31 May 2024.^12^ The CFR significantly exceeded the WHO-recommended case fatality threshold of under 1%.^13^ The national attack rate stood at 114 cases per 100 000 population, underscoring the outbreak’s extensive reach. As presented in table 1, Lusaka Province bore the greatest burden, accounting for 76% of cases (17 809) and 77% of deaths (570), with a concerning 74% of deaths (322) occurring in the community. Copperbelt Province, the second-most affected region, reported 2126 cases and 69 deaths, with Ndola, Kitwe and Chililabombwe districts most severely impacted, as detailed in table 2.^12 14^

**Table 1:** Provincial distribution of cumulative cholera cases, deaths, discharges and incidence rates during the October 2023–June 2024 outbreak in Zambia

Province	Cumulative cases	Total deaths	Health facility deaths	Community deaths	Deaths (%)	Health facility deaths (%)	Incidence rate
Lusaka	17 805	570	248	322	3.2	1.4	560.7
Copperbelt	2126	69	26	43	3.2	1.2	74.0
Central	1749	55	19	36	3.1	1.1	74.1
Southern	917	21	4	17	2.3	0.4	37.2
Eastern	372	9	3	6	1.9	0.7	18.2
North-Western	237	15	4	11	6.3	1.8	17.7
Western	40	1	0	1	2.5	0.0	2.8
Northern	23	0	0	0	0.0	0.0	1.4
Muchinga	7	0	0	0	0.0	0.0	0.7
Luapula	1	0	0	0	0.0	0.0	0.1
Total	23 277	740	304	436	3.2	1.3	114.0

**Table 2:** District-level breakdown of cholera cases, deaths and discharges in Copperbelt Province during the October 2023–June 2024 outbreak

District	Cum. suspected cases	Cum. culture conf. cases	Cum. RDT positive	Cum. deaths
Chililabombwe	84	9	34	3
Chingola	6	2	4	0
Kitwe	671	6	119	16
Mufulira	26	3	14	2
Ndola	1291	14	177	46
Luanshya	11	3	4	0
Masaiti	18	6	11	0
Kalulushi	7	4	7	1
Lufwanyama	5	1	4	1
Mpongwe	7	1	3	0
Total	2126	49	377	69

ConfConfirmedCumCummulativeRDTRapid Diagnistic Test

**Figure 1:**
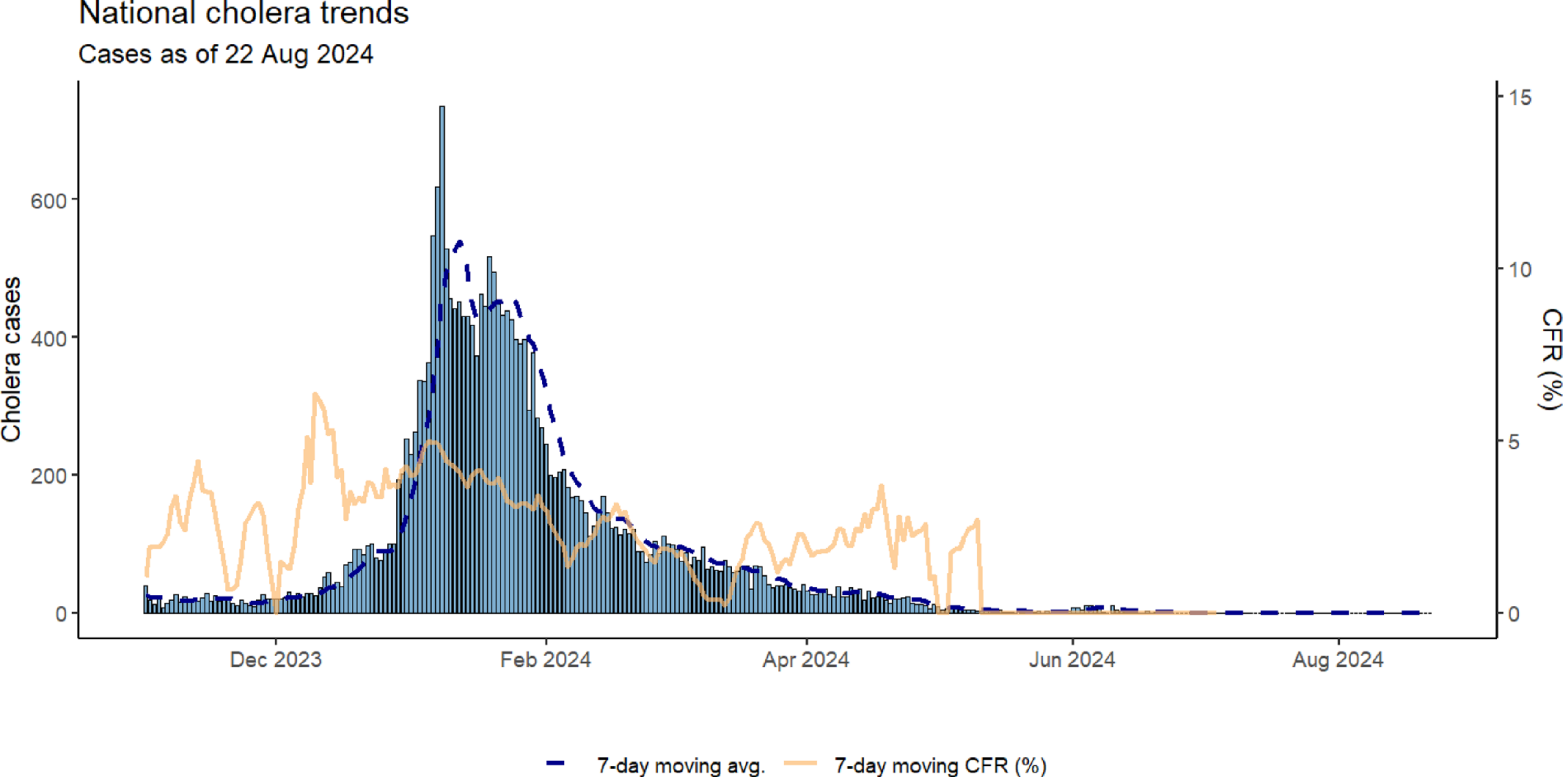
National cholera trends in Zambia, October 2023–June 2024, showing daily cases (bars), 7-day moving average of cases (dashed blue line) and 7-day moving average case fatality rate (CFR, solid yellow line).

National trends in cholera cases and the 7-day moving average CFR, illustrated in figure 1, highlight the outbreak’s progression and severity. Cases peaked in February 2024, with a subsequent decline by June 2024. However, the CFR (yellow line) consistently remained above the 1% threshold, indicating persistent challenges in case management and healthcare access.

Table 1 provides a breakdown of cumulative cases, deaths, discharges and incidence rates across the provinces, underscoring the disparity in cholera’s impact. Lusaka Province recorded the highest incidence rate (560.7 per 100 000 population), followed by Copperbelt (74.0 per 100 000) and Central (74.3 per 100 000). Notably, North-Western Province reported the highest CFR (6.3%), attributed to challenges in healthcare access and delayed treatment. Table 2 focuses on district-level data within Copperbelt Province, with Ndola accounting for over 60% of cases and 67% of deaths in the province. These data reflect significant gaps in surveillance, healthcare delivery and community engagement.

Despite putting in a lot of efforts to control the outbreak, the number of cases continued to increase with a very high proportion of community deaths in Lusaka (322, 74%) and Copperbelt (43, 62%) provinces clearly highlighting a gap in community interventions. Evidence from several studies suggests that interventions for effective cholera control and response epidemics should focus on community-centred approaches that involve a combination of water, sanitation and hygiene (WaSH), effective and rapid surveillance, enhanced social mobilisation and timely and appropriate case management. Based on this evidence, the Ministry of Health and the Zambian National Public Health Institute and with support from WHO, devised and implemented the ICSCC in the three most affected districts of the Copperbelt Province (Ndola, Kitwe and Chililabombwe).

This article describes the implementation of the ICSCC, analysing its effectiveness in addressing WASH, surveillance, social mobilisation and quality of healthcare gaps. The findings aim to provide a scalable model for managing similar public health crises in resource-constrained settings.

### The Integrated Community Strategy for Cholera Control (ICSCC) approach

The 2023–2024 cholera outbreak in Zambia highlighted a critical need for effective community-level interventions to facilitate early detection and management of cases, promote preventive behaviours and address underlying social determinants of health such as access to safe water and sanitation. Addressing cholera in Zambia requires a multifaceted approach, including improving access to safe water, sanitation and hygiene (WASH), strengthening surveillance, case management and community engagement efforts.^11 15^ The Zambia National Cholera Elimination Plan^11^ aligned to the Ending cholera: a Global Roadmap to 2030^16^ emphasises a comprehensive and multisectoral approach encompassing early detection, rapid response, multisectoral coordination, community-based operational planning and strategic objectives focusing on control measures. An example of the operationalisation of the MCEP is the ICSCC, which is designed to implement the MCEP at the community level.

ICSCC is a comprehensive public health approach that places communities at the centre of disease management efforts. The strategy integrates disease surveillance, case management, risk communication, community engagement, infection prevention and control and WASH initiatives. ICSCC aims to reduce mortality and limit disease spread through enhanced surveillance, prompt treatment access, robust prevention measures, active community involvement and improved sanitation practices. This approach emphasises early detection, rapid response, enhanced information, education and communication and multisectoral coordination to effectively manage and control disease outbreaks at the community level.

### Implementation process

The implementation of the ICSCC in the Copperbelt Province of Zambia followed a structured and strategic approach to ensure the effective delivery and sustainability of interventions. The process began with detailed planning and site selection, where cholera hotspots were identified using epidemiological data and situation maps. This analysis highlighted Ndola, Kitwe and Chililabombwe as priority areas due to their high incidence rates and dense populations. Stakeholders, including provincial and district health authorities, were engaged early to secure their buy-in and ownership of the strategy. Budgeting and resource allocation were meticulously planned to cover personnel allowances, transportation, supplies and operational costs, while a clear timeline for implementation was established to monitor progress and evaluate outcomes.

Community-based volunteers (CBVs) and healthcare workers were identified and trained across the three districts. Training sessions, supported by WHO and partners such as the Red Cross, MSF and Amref, equipped participants with skills in surveillance, case management, risk communication, infection prevention and control, WASH interventions and digital reporting tools. Altogether, 80 CBVs and 85 healthcare workers were trained and supported with essential resources, including liquid and granular chlorine, cholera test kits, public health education materials and sanitation items. Transportation logistics were also organised to ensure timely delivery of supplies and facilitate the mobility of technical teams to affected areas.

The deployment of interventions involved assigning multidisciplinary teams to the targeted communities. Community-based teams, consisting of five trained members per community, were formed to carry out field activities such as door-to-door sensitisation campaigns. These campaigns emphasised regular handwashing with soap, safe water use and storage, proper sanitation, safe food handling and prompt treatment-seeking behaviours. Teams also implemented WASH activities such as chlorinating water sources, distributing liquid chlorine and liming latrines to enhance sanitation and hygiene practices.^17^,^19^ Surveillance was another critical component, with community teams identifying and reporting suspected cholera cases to health facilities for confirmation and treatment.

Monitoring and supervision were integral to ensuring the strategy’s effectiveness. Environmental health practitioners from local health facilities closely supervised community teams, providing technical support and ensuring alignment with broader health response frameworks. Regular reporting from CBVs allowed for real-time adjustments to the intervention strategy and allocation of additional resources where needed. At the national level, a dedicated oversight team supported by WHO and other technical partners ensured effective coordination and decision-making across all levels of implementation. This strategic approach not only mitigated cholera cases but also strengthened community resilience, demonstrating the effectiveness of the ICSCC strategy in responding to public health emergencies.

Throughout the process, ethical considerations were prioritised. The activities were conducted as part of an emergency public health response with the approval of local health authorities. Confidentiality and cultural sensitivity were respected in all community interactions, and approval was sought from the National Health Research Authority to publish the findings and best practices arising from the outbreak response.

**Figure 2:**
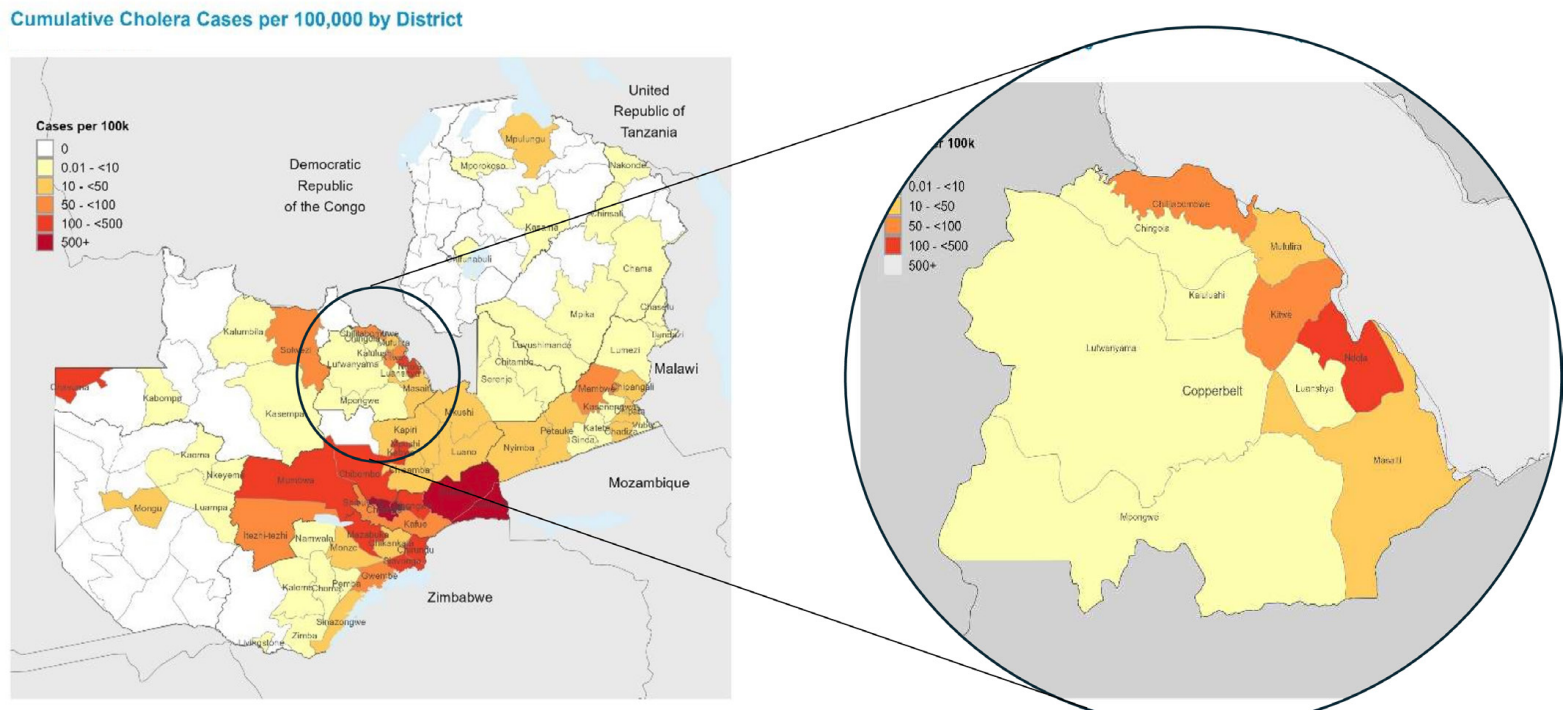
Cumulative cholera cases per 100 000 by district in Zambia (left) and Copperbelt Province (right) as of 18 August 2024, highlighting high-incidence areas such as Ndola, Kitwe and Chililabombwe.

Figure 2 illustrates cumulative cholera cases per 100 000 population by district across Zambia, with a detailed focus on the Copperbelt Province. The map highlights incidence rates, drawing attention to high-burden districts such as Ndola, Kitwe and Chililabombwe.

### Outcomes

**Figure 3:**
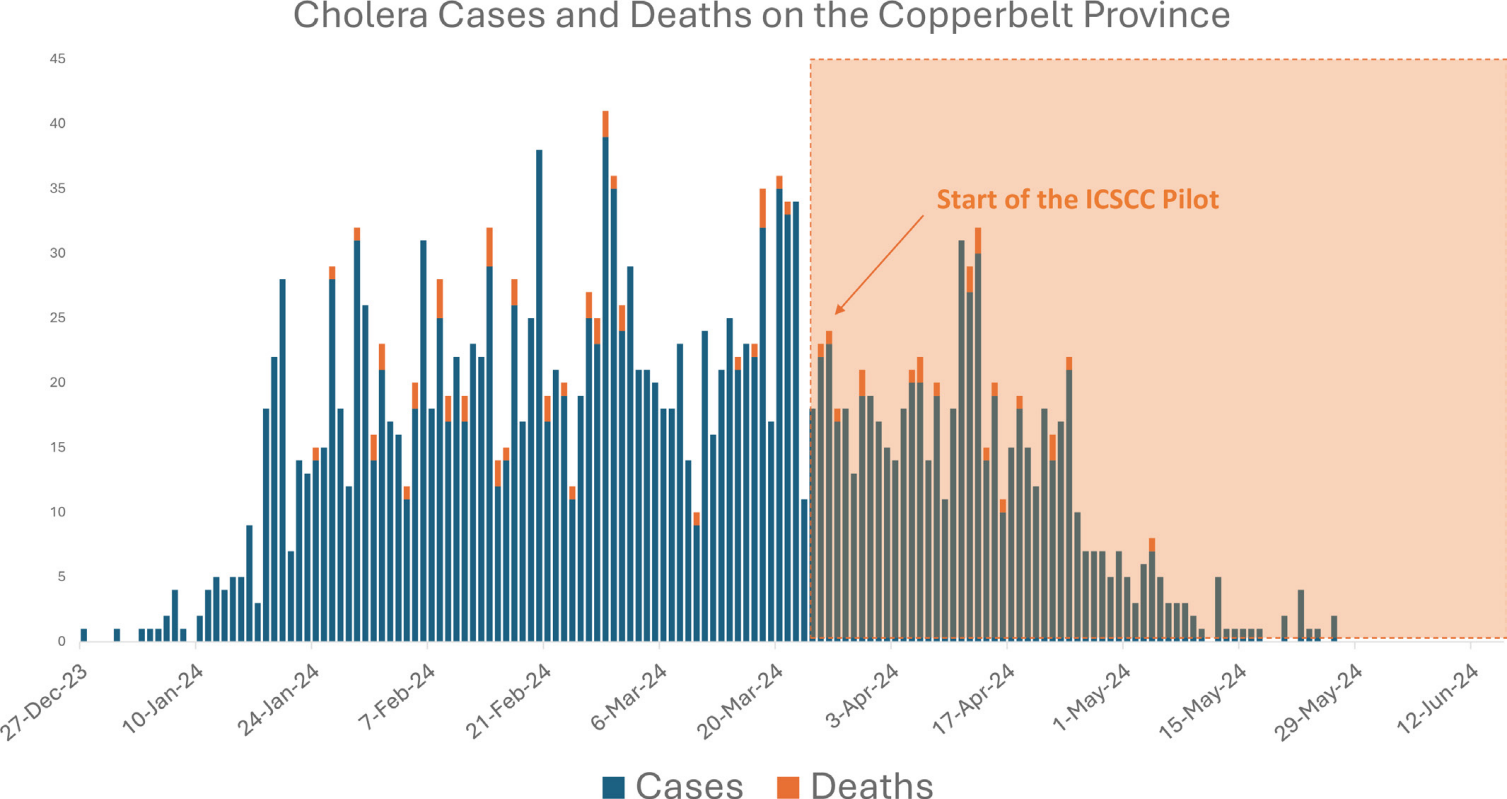
Cholera cases and deaths in the Copperbelt Province

The implementation of the ICSCC in Ndola, Kitwe and Chililabombwe districts was associated with notable improvements in cholera-related indicators in the province. By the end of May 2024, the number of new daily cholera cases had consistently fallen below ten cases per day in both Ndola and Kitwe districts, with several days reporting fewer than five new cases. Concurrently, the number of cholera-related deaths decreased to nearly zero by late April and remained minimal through May. These reductions are visually represented in figure 3, which illustrates a marked decline in both cases and deaths following the initiation of the ICSCC interventions.

In addition to the epidemiological outcomes, substantial enhancements in WASH practices were observed across the intervention areas. This improvement was primarily driven by the distribution of liquid and granular chlorine, enabling consistent water disinfection. Furthermore, hand hygiene practices saw a notable rise, with regular handwashing with soap becoming more prevalent due to targeted awareness and sensitisation campaigns conducted by community-based volunteers. A community volunteer from the Chipulukusu area in Kitwe noted, ‘Our awareness campaigns on handwashing and food hygiene led to noticeable improvements. Many households now regularly use chlorine for water treatment, which has greatly reduced cholera transmission’. Increased knowledge about cholera prevention and improved hygiene practices were observed among community members. In Chililabombwe, for instance, respondents noted the community’s appreciation and eagerness to learn about prevention measures.

Community engagement activities, including community meetings and multiple clean-up campaigns, fostered a stronger sense of collective responsibility towards cholera prevention. The establishment of an incident management system facilitated effective coordination among health facilities and community teams, ensuring rapid responses to emerging cholera cases. These coordinated efforts contributed to improved surveillance, timely case management and enhanced public health education, collectively strengthening the local response mechanisms.

However, it is important to acknowledge that the observed reductions in cholera cases, and deaths may not be solely attributable to the ICSCC interventions. Other factors, such as the natural waning of the outbreak, seasonal variations affecting cholera transmission and concurrent public health measures^20 21^ not detailed in this study, could have also played significant roles in mitigating the outbreak’s impact.

### Challenges faced

The implementation of ICSCC in Zambia’s Copperbelt Province encountered several challenges and limitations that hindered its effectiveness. Despite the strategy’s success in reducing cholera cases and deaths, resource constraints and logistical issues posed significant barriers. Shortages of essential supplies such as oral rehydration salts (ORS), chlorine and laboratory reagents, and transportation difficulties hindered the timely delivery of interventions and response activities.

While the ICSCC strategy was introduced to enhance community engagement, overcoming deeply rooted challenges required ongoing effort. Initial barriers included mistrust and restricted access to households due to a lack of proper identification for CBVs, as well as entrenched beliefs and behaviours that resisted cholera prevention measures.^22^ Data management deficiencies significantly impacted the efficiency and accuracy of data management, particularly at the community level. Lack of standardised reporting tools and inconsistencies in data flow led to delays in case management and response activities.

Surveillance gaps were another significant challenge. Weaknesses in surveillance and reporting strategies, including inadequate community-level data collection, led to potential delays in case management and response activities. Health facilities faced significant strain, particularly during early morning hours, leading to a high number of deaths due to inadequate case management and dehydration treatment. Effective coordination and information flow among various stakeholders and intervention points were lacking, resulting in delayed responses. The need to tailor interventions to address localised risk factors and contextual challenges, such as erratic water supply, was recognised as crucial.

### Lessons learned

The lessons learnt from the implementation of the ICSCC underscore the potential of integrated community-based approaches in managing cholera outbreaks and enhancing public health preparedness. The ICSCC demonstrated that a well-coordinated multisectoral approach is crucial for effective cholera control. The integration of surveillance, case management, WASH interventions and community engagement proved more effective than isolated interventions. Early engagement of community leaders and volunteers was critical in building trust, disseminating information and ensuring the adoption of preventive measures. Sustained community involvement throughout the response helped maintain momentum and reinforce positive behaviours. The success of the ICSCC was largely due to its flexibility in adapting to local contexts. Tailoring interventions to address specific risk factors and socio-cultural dynamics in each community enhanced their relevance and effectiveness. Investing in training and capacity building for local health workers and community volunteers not only improved the immediate response but also built a foundation for long-term resilience against future outbreaks.^23 24^

### Recommendations for policy and practice

The experience of implementing the ICSCC highlights several recommendations that Zambia’s cholera control policies can adopt to build resilience and sustainably prevent future outbreaks. Key recommendations for policy and practice include:

1. Sustaining Health Education and Behavioural Change Campaigns: Ongoing health education and community-led water, sanitation and hygiene (WASH) committees are essential for reinforcing hygiene practices. Regular simulation exercises and refresher training for health workers and community volunteers should be institutionalised to maintain preparedness.
2. Integrating WASH and Sustainable Logistics: Sustainable cholera control requires embedding WASH initiatives into community practices, such as point-of-collection chlorination, regular distribution of chlorine and household-level safe water messaging. Effective logistics, including well-managed stockpiles of ORS and other essentials, ensure continuous cholera prevention efforts and timely response.
3. Strengthening Surveillance and Early Warning Systems: Enhancing disease surveillance and environmental monitoring enables prompt detection and reporting, particularly in high-risk areas. Training community-based volunteers and healthcare workers in case detection and reporting is critical to the ICSCC’s success.
4. Fostering Community Engagement and Socioeconomic Support: Community engagement is crucial for maintaining public health gains. ICSCC should continue to involve local leaders and community members in hygiene campaigns and safe waste disposal practices. Addressing broader socioeconomic factors, such as access to safe food and water and promoting gender equity, further strengthens cholera resilience.
5. System-Wide Health and Emergency Preparedness: Cholera control efforts should be integrated into broader health system initiatives to ensure comprehensive emergency preparedness. Strengthening primary healthcare, enhancing surveillance capabilities and fostering collaboration between stakeholders reinforce these goals.
6. Operationalising the Zambia Multisectoral Cholera Control Plan (NMCP): The ICSCC offers a practical model for NMCP implementation by translating national objectives into community-level actions. This involves tailored interventions that respond to specific local needs and reinforce coordination between government, health institutions and communities.

Through these integrated strategies, Zambia can reinforce community resilience, address social determinants of health and adopt a proactive, system-wide approach to cholera prevention and control, setting a robust foundation for enduring public health improvements.^25 26^

## Conclusion

The implementation of the ICSCC in Zambia’s Copperbelt Province demonstrated promising outcomes in reducing cholera incidence and mortality, enhancing WASH practices and strengthening community resilience. While the ICSCC interventions—encompassing enhanced surveillance, community engagement and improved sanitation practices—likely contributed to the observed decline in cholera cases and deaths, it is essential to consider the influence of other mitigating factors such as the natural progression of the outbreak and concurrent public health initiatives.

This case study underscores the potential of integrated, community-based approaches in managing cholera outbreaks and building long-term public health resilience. The success of the ICSCC highlights the importance of robust stakeholder coordination, early community involvement and the adaptability of interventions to local socio-cultural contexts. These findings align with existing literature that emphasises the multifaceted nature of effective cholera control strategies.^25 27 28^

However, establishing a definitive causal relationship between ICSCC interventions and the reduction in cholera cases requires more robust epidemiological studies with comprehensive data analysis. Future research should aim to employ rigorous study designs to isolate the impact of specific interventions within integrated strategies.

In conclusion, the ICSCC serves as a scalable and adaptable model for cholera control in resource-constrained settings, offering valuable insights for policymakers and public health practitioners. By leveraging community engagement and ensuring comprehensive WASH interventions, similar strategies can be effectively employed to mitigate cholera outbreaks and enhance public health preparedness in other regions facing comparable challenges.

## Data Availability

Data sharing not applicable as no datasets generated and/or analysed for this study.
